# 106 Adipose Derived Stem Cell Paracrine Factor Release in Smokers vs Non Smokers with Burn Injuries

**DOI:** 10.1093/jbcr/irad045.079

**Published:** 2023-05-15

**Authors:** Paige Deville, Alison Smith, Herbert Phelan, Jeffrey Carter, Olivia Warren, Cameron Fontenot

**Affiliations:** LSUHSC New Orleans Department of General Surgery, New Orleans, Louisiana; LSU General Surgery, New Orleans, Louisiana; LSUHSC New Orleans Department of General Surgery, New Orleans, Louisiana; LSUHSC New Orleans Department of General Surgery, New Orleans, Louisiana; LSUHSC, New Orleans, Louisiana; LSUHSC, New Orleans, Louisiana; LSUHSC New Orleans Department of General Surgery, New Orleans, Louisiana; LSU General Surgery, New Orleans, Louisiana; LSUHSC New Orleans Department of General Surgery, New Orleans, Louisiana; LSUHSC New Orleans Department of General Surgery, New Orleans, Louisiana; LSUHSC, New Orleans, Louisiana; LSUHSC, New Orleans, Louisiana; LSUHSC New Orleans Department of General Surgery, New Orleans, Louisiana; LSU General Surgery, New Orleans, Louisiana; LSUHSC New Orleans Department of General Surgery, New Orleans, Louisiana; LSUHSC New Orleans Department of General Surgery, New Orleans, Louisiana; LSUHSC, New Orleans, Louisiana; LSUHSC, New Orleans, Louisiana; LSUHSC New Orleans Department of General Surgery, New Orleans, Louisiana; LSU General Surgery, New Orleans, Louisiana; LSUHSC New Orleans Department of General Surgery, New Orleans, Louisiana; LSUHSC New Orleans Department of General Surgery, New Orleans, Louisiana; LSUHSC, New Orleans, Louisiana; LSUHSC, New Orleans, Louisiana; LSUHSC New Orleans Department of General Surgery, New Orleans, Louisiana; LSU General Surgery, New Orleans, Louisiana; LSUHSC New Orleans Department of General Surgery, New Orleans, Louisiana; LSUHSC New Orleans Department of General Surgery, New Orleans, Louisiana; LSUHSC, New Orleans, Louisiana; LSUHSC, New Orleans, Louisiana; LSUHSC New Orleans Department of General Surgery, New Orleans, Louisiana; LSU General Surgery, New Orleans, Louisiana; LSUHSC New Orleans Department of General Surgery, New Orleans, Louisiana; LSUHSC New Orleans Department of General Surgery, New Orleans, Louisiana; LSUHSC, New Orleans, Louisiana; LSUHSC, New Orleans, Louisiana

## Abstract

**Introduction:**

Adipose-derived stem cells (ADSCs) have an important role in the modulation of burned tissue repair through the release of paracrine factors that stimulate the wound healing response. In this study, we tested the hypothesis that smoking status alters the profile of paracrine factors secreted from ADSCs isolated from damaged adipose tissue.

**Methods:**

Adipose tissue was collected from adult patients (N=8) with severe burn injuries ( >20% total body surface area) at the index operation. ADSCs were extracted and cultured *in vitro*. Supernatants were harvested 30 hours after plating and used for cytokine determinations by Multiplex assay. Fluorescence activated single cell sorting (FACS) confirmed their phenotype with markers CD 90, CD 166, and CD 73. Univariate analyses were performed to compare the two cohorts (Smokers vs non smokers). All outliers were removed.

**Results:**

Higher amounts of anti-inflammatory cytokines IL-4(p=0.03) and IL-10(p=0.04) and pro-inflammatory cytokines TNF-alpha (0.03), IL-8, and IFN-gamma (p=0.03) were detected in burn patients who were current everyday smokers when compared to nonsmokers, or former smokers. No significant differences in supernatant concentrations of IL-17, IL-1 beta, TGF-alpha, IL-6, and IL-13 were observed (p >0.05). Mortality was higher in the smoker group when compared to non-smokers (p=0.02).

**Conclusions:**

The results from this study suggest that smoking status in patients with a major burn injury may alter the profile of paracrine factors secreted from ADSCs, and on-going studies will increase sample size and refine experimental approach. Furthermore, these results support the need for studies examining the systemic effects of smoking status of patients suffering burn injuries impacts the wound healing.

**Applicability of Research to Practice:**

Study the effects of smoking on wound healing in burn injuries.

